# Assessing the diagnostic accuracy of routine hematoxylin and eosin, Alcian Blue/Periodic Acid-Schiff, and Giemsa stains in the detection of *Helicobacter pylori* in gastric biopsies

**DOI:** 10.12688/f1000research.170290.2

**Published:** 2026-01-10

**Authors:** Nasar Alwahaibi, Al-Mutaz Al Mamari, Arwa Al Aamri, Yaqeen Al Sulimani, Alwaleed Al Balushi

**Affiliations:** 1 Department of Biomedical Science, Sultan Qaboos University College of Medicine and Health Science, Muscat, 123, Oman

**Keywords:** Helicobacter pylori, gastric biopsy, Giemsa stain, hematoxylin and eosin, Alcian Blue/Periodic Acid-Schiff, sensitivity, specificity

## Abstract

**Background:**

Hematoxylin and Eosin (H&E), Alcian Blue/Periodic Acid-Schiff (AB/PAS), and Giemsa stains are routinely used in the histopathological evaluation of gastric biopsies. However, comparative data on their diagnostic performance and cost-effectiveness in detecting *Helicobacter pylori* are limited. This study aimed to assess the feasibility of using H&E and AB/PAS as alternatives to Giemsa.

**Methods:**

A retrospective study was conducted on 816 gastric biopsy cases collected between 2019 and 2021. Three slides (H&E, Giemsa, and AB/PAS) were previously prepared from each paraffin-embedded tissue sample and blindly evaluated by three independent examiners. Sensitivity, specificity, positive predictive value (PPV), negative predictive value (NPV), and overall diagnostic accuracy were calculated using 2×2 contingency tables.

**Results:**

>H&E yielded a sensitivity of 51.6%, specificity of 74.4%, and diagnostic accuracy of 66.4% when compared to Giemsa as the reference standard. In comparison, AB/PAS exhibited a sensitivity of 45.9%, specificity of 73.2%, and an accuracy of 63.7%. When evaluating operational factors, H&E emerged as the most cost-effective and fastest method, while AB/PAS was associated with higher costs and longer processing times. Overall, Giemsa consistently demonstrated superior diagnostic performance across the assessed metrics, positioning it as the more reliable choice for *H. pylori* detection within this comparative framework.

**Conclusions:**

H&E shows potential for initial screening but its limited sensitivity prevents definitive diagnosis. AB/PAS proved less effective and economical. Giemsa maintained superior diagnostic performance. This study provides a comparative methodological assessment, suggesting H&E and Giemsa combinations could enhance detection efficiency and accuracy. Further prospective validation is needed before clinical implementation.

## Introduction

*Helicobacter pylori* ( *H. pylori*) is a significant global health concern, with nearly half of the world’s population infected by this bacterium. ^1^ Beyond its role in chronic gastritis and peptic ulcers, *H. pylori* is a causal driver of gastric carcinogenesis, profoundly influencing disease progression and clinical outcomes. ^2^ This gram-negative bacterium colonizes the gastric mucosa and plays a pivotal role in the pathogenesis of various gastric disorders. For instance, studies have shown that *H. pylori* infection can facilitate cell migration and impact clinical outcomes in gastric cancer, underscoring its relevance beyond mere infection detection. ^3^ Given its deep connection to cancer development, accurate identification of *H. pylori* is critical in clinical practice, impacting both diagnostic and prognostic decisions. Histopathological demonstration, due to its high sensitivity and specificity and additional morphological information, remains one of the most reliable methods for detecting *H. pylori*, particularly in gastric biopsy specimens. ^4^ Therefore, studying *H. pylori* detection methods is essential not only for improving diagnostic accuracy but also for better understanding its pathogenic mechanisms and developing effective treatment strategies to mitigate its widespread impact on public health and specifically, its contribution to gastric cancer.

Hematoxylin and Eosin (H&E), Alcian Blue/Periodic Acid-Schiff (AB/PAS), and Giemsa staining methods are essential routine stains for the histopathological evaluation of gastric biopsies. A survey was conducted into the use of H&E and special stains on gastric specimens in histopathology departments within the National Health Service, United Kingdom. One hundred and sixty-seven histopathology departments in the UK were contacted using an e-mail questionnaire. Gastric specimens are stained using H&E in 47% of departments and 53% use H&E combined with special stains (AB/PAS, and Giemsa). ^5^

These stains play a critical role in diagnosing various gastric conditions, including the identification of *H. pylori.* H&E provides an overall assessment of tissue architecture, AB/PAS highlights mucin alterations, and Giemsa specifically stains *H. pylori*, making them complementary tools in gastric pathology. Giemsa staining is widely regarded as a specific, cheap, easy to perform and reliable method for detecting *H. pylori* in gastric biopsies. Its ability to clearly highlight the bacterium against the background tissue makes it a preferred choice in gastric pathology. ^6– 9^ However, its limitation lies in its specificity, as it is not suitable for general histopathological evaluation. H&E is the most commonly used routine stain in histopathology due to its ability to provide excellent contrast between cellular and extracellular structures, making it a valuable tool for general tissue examination. Certain laboratories can optimize H&E staining to enhance the detection of *H. pylori* in gastric biopsies. ^10, 11^ However, its lack of specificity in differentiating between certain cellular components or microorganisms is a notable limitation. AB/PAS is primarily employed to differentiate between acidic and neutral mucins in the gastric epithelium. Periodic Acid-Schiff (PAS) reaction targets 1,2-glycol groups present in carbohydrates, oxidizing them to form aldehydes, which then react with Schiff’s reagent to produce a magenta color. ^12^ The outer membrane of *H. pylori* contains carbohydrate-rich structures, such as polysaccharides and glycans, that include 1,2-glycol groups. ^13, 14^ However, the amount and accessibility of 1,2-glycol groups in *H. pylori* may vary among strains, this will affect the consistency of detection with PAS. If its utility in identifying *H. pylori* can be established, it might serve as an adjunct to specific staining methods like Giemsa, providing broader pathological insights.

Although these staining techniques are widely used, there is a notable lack of comprehensive studies comparing their diagnostic efficacy, accuracy, staining time, and cost-effectiveness. This gap in the literature highlights the need for further investigation. This study seeks to address this by evaluating the strengths and limitations of H&E, AB/PAS, and Giemsa stains in gastric biopsies. Therefore, this study aimed to evaluate the feasibility of utilizing H&E and AB/PAS staining methods as potential alternatives to the Giemsa staining method for the detection of *H. pylori* in gastric biopsies.

## Materials and methods

### Study setting

This retrospective cohort study was conducted in the Pathology Department, Sultan Qaboos University Hospital, utilizing gastric biopsy cases collected between 2019 and 2021. The data were accessed for research purposes on June 11, 2024. Demographic data for the selected cases were obtained from the Trak-care system. The slides were stored in appropriate boxes at room temperature, ensuring they were not exposed to sunlight or humidity to preserve their quality.

### Sample selection

The sample selection was based on inclusion and exclusion criteria to ensure the reliability of the comparative analysis. To be included in the study, each case was required to have a complete set of slides stained with the three selected methods: H&E, Giemsa, and AB/PAS. Cases were excluded if they lacked any one of the required stained slides, were repeated submissions of the same patient (duplicates), or involved non-gastric biopsy specimens, as the study focused exclusively on gastric tissue. Out of the initial pool of 1,370 cases reviewed, 544 did not meet the inclusion criteria and were excluded. Therefore, a total of 816 cases with complete and eligible staining data were included in the final analysis.

### Cost and staining procedures

As this was a retrospective study, three glass slides were previously prepared from each paraffin-embedded gastric tissue biopsy. Each slide was stained using one of the three standard histological methods: H&E, Giemsa, or AB/PAS. For H&E, sections were stained with Harris hematoxylin (300 mL per staining dish; Cellavision, Cat# 361075) for 8 minutes at room temperature, followed by eosin Y (300 mL per staining dish; Surgipath, Leica Biosystems, Cat# 3801619) for 4 minutes at room temperature. The total staining time was approximately 15–25 minutes. Reagent costs for both dyes were obtained from local suppliers. For Giemsa, sections were stained with Giemsa working solution prepared in pH 6.8 buffer (single-slide dish, 50 mL; Merck, Sigma-Aldrich, Cat# GS500) for 25 minutes at room temperature. The procedure required approximately 30–45 minutes. For AB/PAS, sections were sequentially stained with 1% Alcian blue, pH 2.5 (200 μL per slide; Fluka Analytical, Sigma-Aldrich, Cat# 33864-99-2) for 20 minutes at room temperature; periodic acid (200 μL per slide; Merck, Sigma-Aldrich, Cat# 375810-25 G) for 5 minutes at room temperature; and Schiff reagent (200 μL per slide; Carl Roth, Cat# X900.2) for 8 minutes at room temperature. The full staining process took approximately 40–60 minutes. Costs for Alcian blue, periodic acid, and Schiff’s reagent were obtained from local suppliers. All staining procedures were performed according to standard protocols under identical laboratory conditions.

### Evaluation

Each stain was evaluated by an independent examiner who remained blinded to the other staining results for the same case, thereby minimizing potential observer bias. In cases of diagnostic uncertainty, a thorough examination was performed using an oil immersion objective at 1,000 × magnification. To ensure diagnostic consensus and address challenging interpretations, particularly difficult cases were further reviewed by an expert examiner. Each stained slide was microscopically examined (Nikon, Module Eclipse Ei R, Kanagawa, Japan) to identify the presence of H. pylori.

### Statistical analysis

All statistical analyses were performed using IBM SPSS Statistics 25. (IBM Corp. Released 2023. IBM SPSS Statistics for Windows, Version 25. Armonk, NY: IBM Corp). To evaluate the diagnostic performance of the H&E and AB/PAS staining methods in detecting *H. pylori*, we constructed 2 × 2 contingency tables using the Giemsa stain as the reference standard, given its widely established superior diagnostic performance and specificity for *H. pylori* detection in routine histopathology. From these tables, we calculated sensitivity, specificity, positive predictive value (PPV), negative predictive value (NPV), and overall diagnostic accuracy for each method. In addition, Receiver Operating Characteristic (ROC) curve analysis was conducted to assess and compare the diagnostic performance of the staining techniques. The Area Under the Curve (AUC) was calculated for each method, providing a comprehensive measure that incorporates both sensitivity and specificity across all possible thresholds. AUC values were interpreted as follows: excellent (≥0.90), good (0.80–0.89), fair (0.70–0.79), and poor (<0.70) according to commonly accepted diagnostic performance benchmarks. Differences in AUC values between stains were evaluated using DeLong’s test, with p-values < 0.05 considered statistically significant.

### Ethical considerations

Ethical approval for this study was obtained from the Medical Research Ethics Committee (MREC) at Sultan Qaboos University, Muscat, Oman, under study number 3040. The committee is responsible for overseeing research ethics and ensuring compliance with institutional and national standards. All procedures involving human participants, materials, or data were conducted in accordance with national regulations and the principles of the Declaration of Helsinki. As the study involved a retrospective analysis of archived staining slides and associated demographic data, the requirement for informed consent was waived by the ethics committee. During data collection, the authors had access to potentially identifying information through the Trak-care system; however, all data were anonymized before analysis, and no identifiable information is included in the final dataset or publication. Participant confidentiality was strictly maintained throughout the study.

## Results

A total of 816 gastric biopsy cases were included in the study. The gender distribution was nearly equal, with 417 (51.1%) males and 399 (48.9%) females. The majority of patients were between 41–60 years of age (36.9%), followed by those aged 61–80 years (29.2%). Younger age groups, including 1–20 years and 21–40 years, represented 9.8% and 21.7% of the cases, respectively, while only 2.5% were in the 81–88 age range. Chronic gastritis was the most frequently observed condition, present in 590 cases (72.3%). Intestinal metaplasia was identified in 162 cases (19.9%), while lymphoplasmacytic infiltrate was found in 304 cases (37.3%). Hyperplastic changes were observed in 102 cases (12.5%). Gastric adenocarcinoma was diagnosed in 25 cases, accounting for 3.1% of the total, and other pathological findings such as inflammatory, regenerative changes, preneoplastic lesions, and neoplastic/tumour-like lesions, were reported in 212 cases (26%) ( Table 1).

**Table 1.** Demographic and histopathological characteristics of the studied gastric biopsy cases.

Demographic characteristics and differential diagnosis	Subcategory	Total (N = 816)
Gender	Male	417 (51.1%)
	Female	399 (48.9%)
Age in Years	1– 20	80 (9.8%)
	21– 40	177 (21.7%)
	41– 60	301 (36.9%)
	61– 80	238 (29.2%)
	81– 88	20 (2.5%)
Chronic gastritis	Presence	590 (72.3%)
	Absence	226 (27.7%)
Intestinal metaplasia	Presence	162 (19.9%)
	Absence	654 (80.1%)
Lymphoplasmacytic infiltrate	Presence	304 (37.3%)
	Absence	512 (62.7%)
Hyperplasia	Presence	102 (12.5%)
	Absence	714 (87.5%)
Gastric adenocarcinoma	presence	25 (3.1%)
	Absence	791 (96.9%)
Others	Presence	212 (26%)
	Absence	604 (74%)

When compared to Giemsa staining, AB/PAS identified 131 of the Giemsa-positive cases as positive, while 142 Giemsa-negative cases were also marked as positive by AB/PAS. Conversely, 154 Giemsa-positive cases were missed (false negatives), and 389 Giemsa-negative cases were correctly identified as negative. For H&E staining, 147 of the Giemsa-positive cases were correctly identified, with 136 false positives. H&E missed 138 Giemsa-positive cases, and 395 Giemsa-negative cases were confirmed as negative. These results indicate that H&E had slightly higher agreement with Giemsa staining than AB/PAS, showing better sensitivity and a slightly higher overall diagnostic concordance in detecting *H. pylori* ( Table 2).

**Table 2.** Comparison of Hematoxylin & Eosin and Alcian Blue/Periodic Acid-Schiff’s Staining Methods with Giemsa for Detection of *H. pylori.*

Stain method	Giemsa Positive	Giemsa Negative	Total
AB/PAS Positive	131	142	273
AB/PAS Negative	154	389	543
H&E Positive	147	136	283
H&E Negative	138	395	533

H & E: Hematoxylin and Eosin, AB/PAS: Alcian Blue/Periodic Acid-Schiff.

Consistent with our primary objective of assessing potential alternatives, the sensitivity, specificity, positive predictive value (PPV), negative predictive value (NPV), and diagnostic accuracy (DA) of H&E and AB/PAS staining methods were systematically compared against Giemsa staining, which served as our reference standard for detecting *H. pylori* in gastric biopsies. H&E staining demonstrated a sensitivity of 51.6%, specificity of 74.4%, PPV of 51.9%, NPV of 74.1%, and an overall diagnostic accuracy of 66.4%. In contrast, AB/PAS showed a slightly lower performance, with a sensitivity of 45.9%, specificity of 73.2%, PPV of 47.9%, NPV of 71.6%, and diagnostic accuracy of 63.7% ( Table 3).

**Table 3.** Comparison of Hematoxylin & Eosin and Alcian Blue/Periodic Acid-Schiff’s diagnostic performance against Giemsa for *H. pylori* detection.

Parameters	H&E	AB/PAS
Sensitivity	51.6%	45.9%
Specificity	74.4%	73.2%
Positive Predictive Value	51.9%	47.9%
Negative Predictive Value	74.1%	71.6%
Diagnostic Accuracy	66.4%	63.7%

H & E: Hematoxylin and Eosin, AB/PAS: Alcian Blue/Periodic Acid-Schiff.

**Figure 1.**
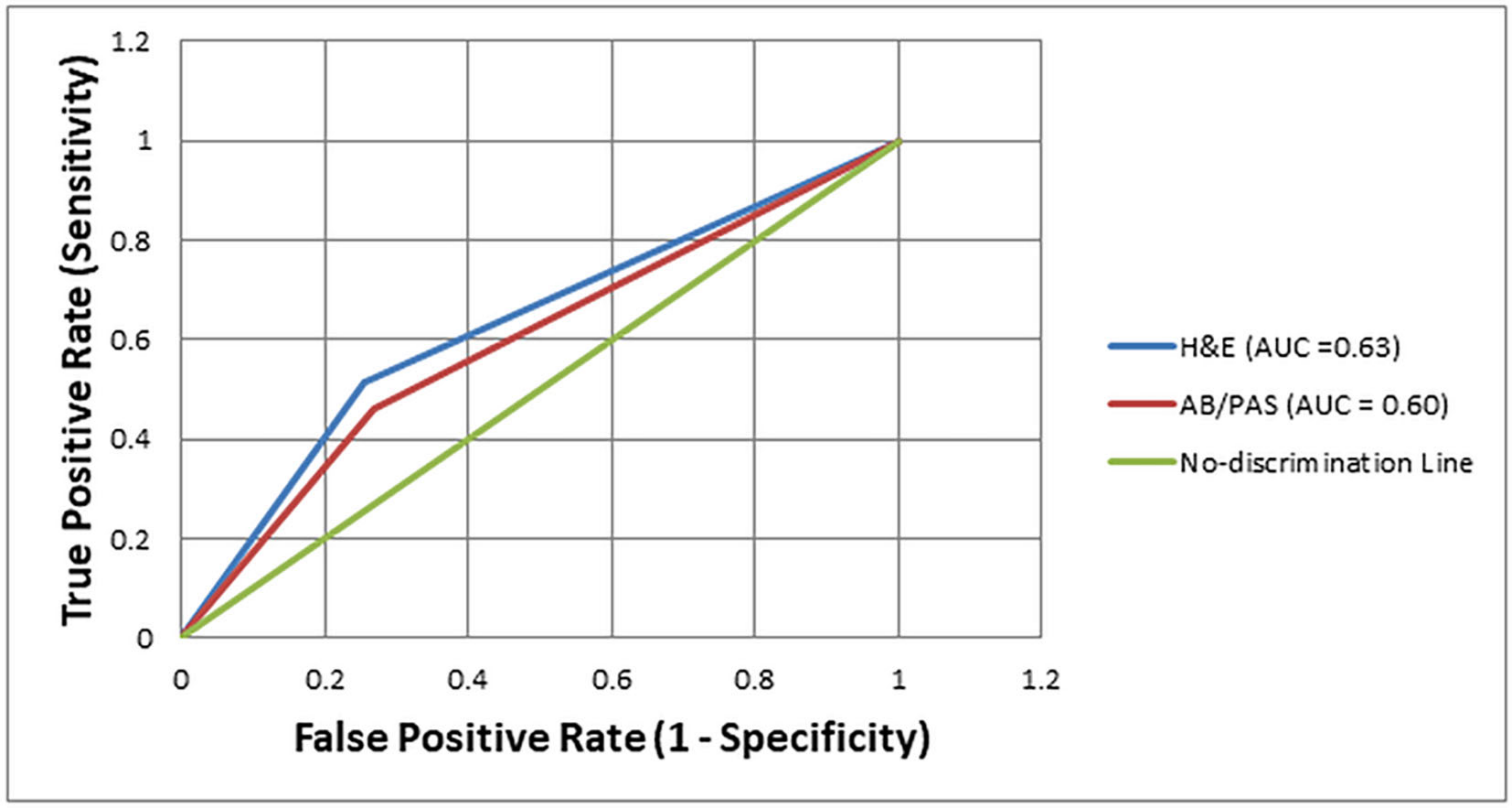
Receiver operating characteristic curve analysis for comparing the diagnostic accuracy of Hematoxylin and Eosin, Alcian Blue/Periodic Acid-Schiff, staining methods for the detection of *H. pylori*, using Giemsa staining as reference.

**Figure 2.**
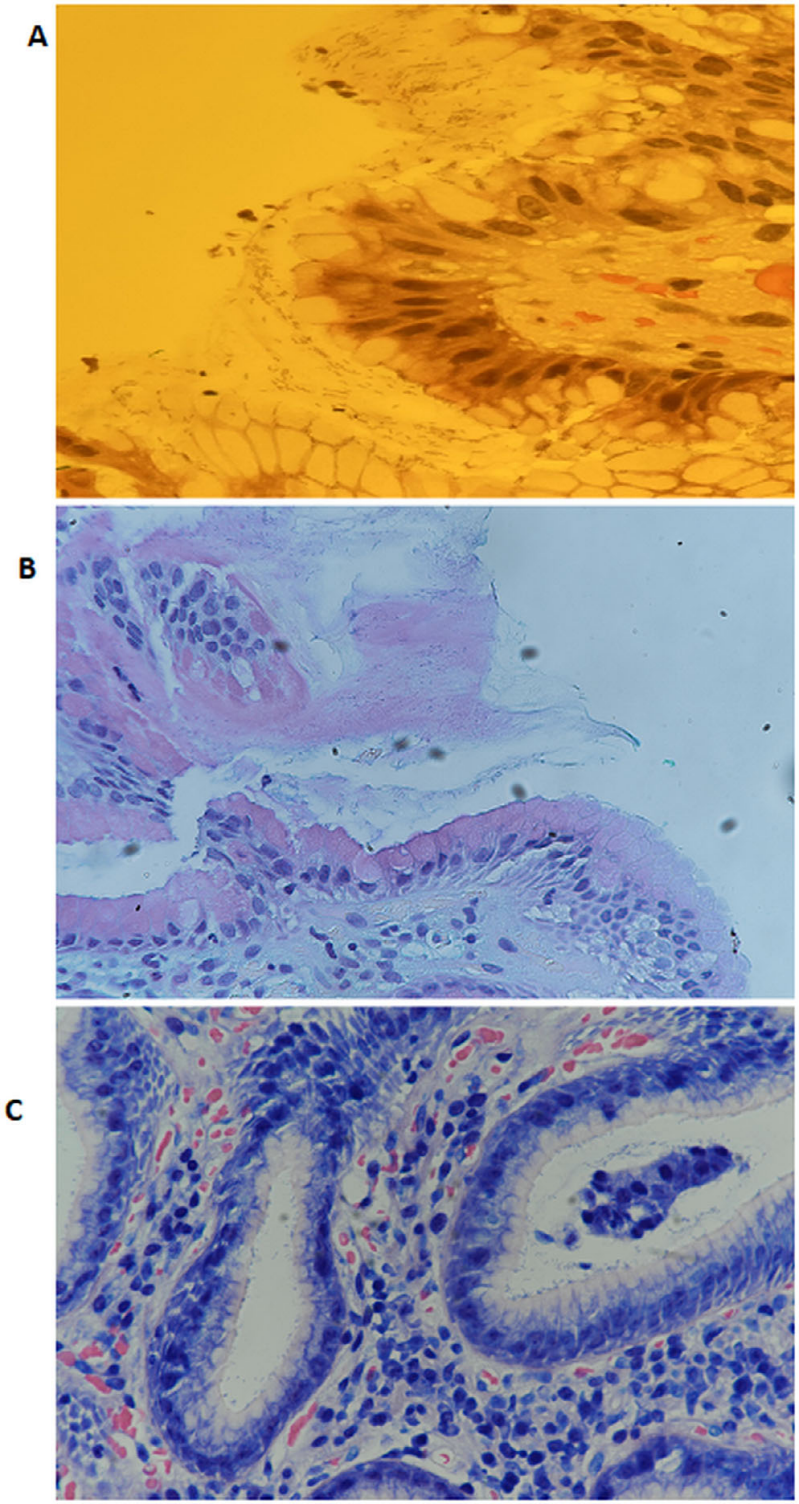
Representative images showing the presence of *H. pylori* in stained gastric tissue sections using Hematoxylin and Eosin (A), Alcian Blue/Periodic Acid-Schiff (B), and Giemsa stain (C) at 40x magnification.

The diagnostic performance of H&E and AB/PAS staining methods for detecting *H. pylori* was further evaluated using Receiver Operating Characteristic (ROC) curve analysis, with Giemsa staining as the reference standard. The Area Under the Curve (AUC) for H&E was 0.63, indicating modest discriminatory ability. In comparison, the AB/PAS stain showed a slightly lower AUC of 0.60, reflecting limited diagnostic performance. Both AUCs positioned only slightly above the no-discrimination line (AUC = 0.50), indicating that while H&E offered a slight improvement over AB/PAS, neither stain alone reached a level indicative of high diagnostic accuracy [ Figure 1]. This underscores the limitations of using these methods as standalone diagnostic tools for *H. pylori* detection. Figure 2 shows representative images of *H. pylori* identified in gastric tissue sections stained with H&E, AB/PAS, and Giemsa.

When demonstrating *H. pylori* in paraffin-embedded gastric tissue sections, H&E is the most cost-effective method, with an estimated reagent cost of USD 0.10–0.20 per slide. This is due to its routine use and reliance on inexpensive, ready-to-use reagents. Giemsa stain has a moderately higher cost, ranging from USD 0.10–0.25 per slide. In contrast, the AB/PAS method is the most expensive, with a per-slide cost of USD 0.45–0.95, primarily due to the use of multiple high-cost reagents such as Alcian Blue and Schiff’s reagent. In terms of time efficiency, H&E also has the shortest staining time, requiring approximately 15 – 25 minutes. Giemsa takes moderately longer, around 30 – 45 minutes, due to preparation and staining steps. AB/PAS is the most time-consuming, taking approximately 40 – 60 minutes because of its multi-step protocol and multiple reagent incubations ( Table 4).

**Table 4.** Comparison cost and staining time for Hematoxylin & Eosin, Giemsa, and Alcian Blue/Periodic Acid-Schiff’s stains.

Stains	Reagent/Stain	Dilution used and bottle size	Cost in OMR and USD	Estimated cost per slide in OMR and USD	Estimated staining time in minutes	Comments
H & E	Harris H	Ready to use, 1000 ml	31.9 **82.75**	0.038-0.077 **0.10 – 0.20**	15 – 25	Lowest cost and fastest method.
	Eosin Y	Ready to use, 500 ml	14.8 **38.40**			
Giemsa Stain	Giemsa	50%, 500 ml	38.9 **101**	0.038 – 0.096 **0.10 – 0.25**	30 – 45	Moderate cost and staining time.
AB/PAS	Alcian Blue 8GX	1%, 25 g	171 **444**	0.17 – 0.37 **0.45 – 0.95**	40 – 60	Highest cost and longest staining time.
	Periodic Acid	1%, 25 g	51.2 **133**			
	Schiff’s Reagent	Ready to use, 1 L	22.1 **57.4**			

OMR: Omani Rials, USD: United Sates Dollars, H & E: Hematoxylin and Eosin, AB/PAS: Alcian Blue/ Periodic Acid-Schiff.

## Discussion

The primary aim of this study was to assess the diagnostic performance, staining efficiency, and cost-effectiveness of H&E and AB/PAS staining methods in comparison to the Giemsa stain, which is commonly regarded as the reference standard for detecting *H. pylori* in gastric biopsy specimens. This multifaceted evaluation was intended to guide laboratories in selecting appropriate staining protocols that balance diagnostic reliability with operational efficiency, particularly in settings with limited resources or high specimen volumes.

The findings highlight that while H&E staining is cost-effective and time-efficient, its diagnostic performance for *H. pylori* is limited. H&E staining demonstrated a relatively low sensitivity of 51.6%, indicating that it correctly identified just over half of the true positive *H. pylori* cases confirmed by Giemsa staining. Its specificity was moderate at 74.4%, reflecting a fair ability to correctly identify negative cases. The positive predictive value of H&E was 51.9%, suggesting that only slightly more than half of the positive results detected by H&E were true positives. Conversely, its negative predictive value was 74.1%, implying a moderate level of confidence in ruling out *H. pylori* when H&E results are negative. The overall diagnostic accuracy of H&E compared to Giemsa was 66.4%, reflecting moderate agreement between the two stains. These findings suggest that H&E staining is more reliable for excluding *H. pylori* rather than confirming its presence and should not be used as a standalone diagnostic tool. Combining H&E with Giemsa may improve detection accuracy in routine practice. Our results are consistent with other studies, such as one from Qassim University, Saudi Arabia, which reported higher sensitivity (66.7%) and specificity (91.2%) though our values were lower. ^15^ Similar findings were also reported. ^6^ In fact, the reported sensitivity of identifying *H. pylori* in gastric specimens using only H&E-stained slides ranges from 66% when evaluated by general histopathologists to as high as 90% when assessed by expert pathologists. ^16, 17^ In addition, *H. pylori* was detected in 37% (14 out of formalin fixed, paraffin wax embedded tissue from 38 gastric biopsy) stained with H&E staining method. ^7^ A study evaluating 325 gastric biopsies from 65 patients with preneoplastic lesions found that H&E had a positivity rate of 41.5% to 49% for detecting *H. pylori*, which was lower than Giemsa staining across all anatomical sites (61.5%–72%). ^18^ Another retrospective study in 390 gastric biopsies showed that H&E staining method has 67% sensitivity in mild gastritis and 83% in moderate or severe gastritis. ^19^

Our findings are in disagreement with other study where they reported that the diagnostic accuracy of H&E was 91.7%, with a sensitivity of 93.2%, and a specificity of 86.7%. However, the gold standard chosen for their study was culture of antral tissue biopsies rather than Giemsa stain. ^20^ A study comparing five staining methods, haematoxylin and eosin (H&E), immunohistochemistry (IHC), silver staining (HpSS), Alcian yellow-toluidine blue (Leung) method (A–Y), and Genta stain, for detecting *H. pylori* in 118 gastric biopsies found no significant difference in the efficacy of H&E, IHC, HpSS, and A–Y. The authors concluded that H&E is sufficient for the initial evaluation of gastric biopsies in patients with upper gastrointestinal symptoms, as it is a well-established, cost-effective, and simple method that offers quick processing and highly reproducible results. ^21^ Another prospective study found that H&E staining is highly effective for identifying *H. pylori* in gastric biopsies. Among 613 biopsies, 71.1% were clearly negative for *H. pylori* on H&E and did not require further staining. Of the inconclusive H&E cases, only a small proportion (15.9%) were toluidine blue positive. In addition, most H&E-positive cases for *H. pylori* were confirmed by toluidine blue staining. The authors concluded that routine use of special stains is unnecessary, as H&E evaluation with selective use of additional stains is sufficient to detect nearly all cases of *H. pylori* gastritis. ^22^

Despite this variability, several studies support the role of H&E as a cost-effective and rapid screening tool. Some research indicates that H&E can achieve higher diagnostic accuracy when assessed by experienced pathologists or when used selectively in combination with other stains like Giemsa or toluidine blue. In particular, studies have shown that special stains may be unnecessary in clear negative or positive cases identified on H&E, supporting a more targeted staining approach in clinical practice. Overall, the collective evidence suggests that while H&E should not be used as a standalone diagnostic tool for *H. pylori*, it remains a valuable first-line method when combined with selective confirmatory staining to enhance diagnostic accuracy and cost-efficiency.

In this study, AB/PAS staining method demonstrated the lowest overall diagnostic performance for *H. pylori* detection among the evaluated techniques. It had the longest staining time (40–60 minutes) and the highest reagent cost (approximately USD 0.45 – 0.95 per slide), largely due to multiple incubation steps involving high-cost reagents. Diagnostic metrics further reflected its limitations: AB/PAS showed a sensitivity of 45.9%, indicating it missed more than half of true positive cases compared to the Giemsa gold standard. The specificity was moderate at 73.2%, reflecting a fair ability to correctly identify negative cases. However, the positive predictive value was relatively low at 47.9%, suggesting a high chance of false positives, while the negative predictive value was 71.6%, showing moderate reliability in ruling out infections. Its overall diagnostic accuracy was only 63.7%, likely influenced by the stain’s tendency to highlight mucins and background components that can obscure the bacteria or mimic its appearance, leading to diagnostic ambiguity.

Supporting evidence from the literature reinforces these findings. For instance, a study, which included forty-five formalin-fixed, paraffin-embedded blocks of gastric biopsies, reported higher sensitivity (81.8%) and specificity (86.9%) for AB/PAS, though still lower than more targeted stains such as Giemsa (reference) and Gimenez stains (88.9% sensitivity and 100% specificity). ^23^ In contrast, another study reported even lower sensitivity (40%) and specificity (67.65%) for AB/PAS, highlighting its inconsistent performance across different studies. ^15^ These discrepancies underline the influence of technique variability and interpretive challenges when using AB/PAS. Overall, despite its routine availability and utility in highlighting mucins and intestinal metaplasia, AB/PAS staining lacks the diagnostic precision needed for reliable detection of *H. pylori.* Its low sensitivity and modest accuracy suggest it should not be used as a standalone method. Instead, it may serve a complementary role alongside more accurate stains like Giemsa, especially in cases requiring concurrent evaluation of gastric mucosal changes. The Giemsa stain is probably one of the most popular stains because of its simplicity and good contrast. ^6, 24– 26^

This study presents several key strengths. It provides a direct comparison of three routinely used histological stains, H&E, Giemsa, and AB/PAS, for detecting *H. pylori*, addressing a notable gap in the existing literature. The inclusion of a large sample size (816 gastric biopsy cases) enhances the statistical validity and generalizability of the results. Furthermore, the study evaluates practical parameters such as staining time and reagent cost, offering important insights into the cost-effectiveness and feasibility of each method in routine histopathology practice.

This study has several limitations. First, the retrospective nature of the study, which relied on histological presentations collected between 2019 and 2021, introduces inherent biases. These include inconsistent data collection, potential slide degradation over time, and variations in staining protocols or processing techniques. Such factors may compromise the reproducibility and reliability of microscopic evaluations. Second, the investigation was confined to a single institution, which is the Sultan Qaboos University Hospital, over a fixed three-year period. This narrow scope raises concerns about selection bias and limits the applicability of the findings to broader clinical settings. Differences in diagnostic protocols, patient demographics, and laboratory practices in other institutions may influence the detection and interpretation of *H. pylori.* Third, while Giemsa staining was used as the reference (gold standard) method, relying on a single stain for validation may introduce classification bias. Giemsa itself may be subject to misinterpretation, especially in cases with low bacterial density, staining artifacts, or faded slides. This may have influenced the perceived diagnostic accuracy of the H&E and AB/PAS methods. Lastly, despite measures taken to reduce observer bias, such as assigning each staining method to a different pathologist and consulting a senior expert for unclear cases, the potential for inter-observer variability in interpreting *H. pylori* presence remains a notable limitation.

The results of this study offer valuable insights primarily for guiding further research and refining existing understanding rather than redefining immediate practice. They can contribute to informing future diagnostic decision-making by clarifying the performance limitations of common staining techniques, thereby supporting pathologists in considering when additional confirmatory testing might be warranted. In clinical practice, these preliminary findings may suggest avenues for exploring more efficient use of existing resources by identifying potential cost-effective screening options. For institutions, these results could serve as a basis for evidence-based discussions regarding potential future revisions to laboratory protocols and staff training programs aimed at enhancing *H. pylori* diagnostic accuracy. Furthermore, this study reinforces the importance of standardized staining procedures to reduce diagnostic variability and improve patient outcomes in future refined protocols.

## Conclusions

While H&E proved to be the most cost-effective and time-efficient stain, its limited sensitivity highlights its inadequacy as a standalone diagnostic method for *H. pylori.* AB/PAS demonstrated the lowest diagnostic accuracy, high cost, and longer staining time, making it less practical for targeted *H. pylori* detection. Giemsa remains the most reliable stain due to its superior sensitivity and specificity. The findings underscore the importance of using Giemsa either alone or in combination with H&E for improved diagnostic confidence. While our findings provide a comparative assessment, it is premature to suggest a definitive change in current practice based solely on this study. However, laboratories could consider exploring H&E's role for initial screening, potentially reserving Giemsa for confirmatory purposes, as a strategy to balance efficiency with diagnostic accuracy in future refined workflows. Crucially, further multi-institutional and prospective research is imperative to rigorously validate these findings, establish clinical utility, and ultimately guide best practices for *H. pylori* detection before any widespread adoption in routine histopathology.

## Reporting guidelines

**Zenodo:** STROBE Statement for Assessing the diagnostic accuracy of routine hematoxylin and eosin, Alcian Blue/Periodic Acid-Schiff, and Giemsa stains in the detection of Helicobacter pylori in gastric biopsies. https://doi.org/10.5281/zenodo.17051552 ^27^

Data are available under the terms of the Creative Commons Attribution 4.0 International license (CC-BY 4.0).

## Data Availability

**Zenodo:** Assessing the diagnostic accuracy of routine hematoxylin and eosin, Alcian Blue/Periodic Acid-Schiff, and Giemsa stains in the detection of Helicobacter pylori in gastric biopsies. https://doi.org/10.5281/zenodo.17330903 ^28^ This project contains the following underlying data:
• Data set for evaluation of *H. pylori* using hematoxylin and eosin, Alcian blue/periodic acid–Schiff, and Giemsa staining in 816 gastric biopsy samples. • Supplementary figure 1. Representative hematoxylin and eosin (H&E)–stained gastric tissue sections (40×) showing the presence and absence of spiral-shaped *Helicobacter pylori*: (A) negative for *H. pylori*; (B) scanty organisms; (C) abundant organisms; (D) highly abundant organisms. • Supplementary figure 2. Representative Alcian blue/periodic acid–Schiff (AB/PAS)–stained gastric tissue sections (40×) showing the presence and absence of spiral-shaped *Helicobacter pylori*: (A) negative for *H. pylori*; (B) scanty organisms; (C) abundant organisms; (D) highly abundant organisms. • Supplementary figure 3. Representative Giemsa-stained gastric tissue sections (40×) showing the presence and absence of spiral-shaped *Helicobacter pylori*: (A) negative for *H. pylori*; (B) scanty organisms; (C) abundant organisms; (D) highly abundant organisms. Data are available under the terms of the Creative Commons Attribution 4.0 International license (CC-BY 4.0).
