# Supplementary material for: Assessing the diagnostic accuracy of routine hematoxylin and eosin, Alcian Blue/Periodic Acid-Schiff, and Giemsa stains in the detection of Helicobacter pylori in gastric biopsies
Source: F1000Res. 2026 Jan 10;14:1148. Originally published 2025 Oct 21. [Version 2] doi: 10.12688/f1000research.170290.2 (PMC12796792; doi:10.12688/f1000research.170290.2)
Supplement: Peer review history

# Reviewer report: Reviewer response for version 2

**DOI:** 10.5256/f1000research.194814.r449128

**Published:** 2026-01-12

**Authors:** Hengrui Liu

**Affiliations:** 1 Guangdong Provincial Key Laboratory of Traditional Chinese Medicine Informatization, Guangzhou, China

The authors have clearly addressed the major issues raised in the earlier round, and the manuscript now read much more smoothly, and the manuscript is now suitable for indexing.

Is the work clearly and accurately presented and does it cite the current literature?

Yes

If applicable, is the statistical analysis and its interpretation appropriate?

Yes

Are all the source data underlying the results available to ensure full reproducibility?

Yes

Is the study design appropriate and is the work technically sound?

Yes

Are the conclusions drawn adequately supported by the results?

Yes

Are sufficient details of methods and analysis provided to allow replication by others?

Yes

Reviewer Expertise:

cancer

I confirm that I have read this submission and believe that I have an appropriate level of expertise to confirm that it is of an acceptable scientific standard.

# Reviewer report: Reviewer response for version 1

**DOI:** 10.5256/f1000research.187732.r435909

**Published:** 2025-12-30

**Authors:** Hengrui Liu

**Affiliations:** 1 Guangdong Provincial Key Laboratory of Traditional Chinese Medicine Informatization, Guangzhou, China

In the abstract, the overall structure work fine but the confidence level feel a bit too high given what actually get tested. Right now it read close to a clinical conclusion, and that make me uneasy. I would really dial back the implication that this approach already meaningfully inform diagnosis or patient outcome, and instead frame it more clearly as a comparative methodological assessment with downstream relevance rather than immediate application. Shortening some claims would help. Just a little restraint here go a long way.

 The background section need more grounding in disease biology and clinical context. Helicobacter pylori is not just a technical detection problem, it is a causal driver of gastric carcinogenesis and disease progression, and that connection should be stated clearly and early so the reader understand why this question matter beyond the lab bench. A brief discussion of how H. pylori infection contribute to gastric cancer development and behavior would strengthen the motivation a lot, and it would be very natural to weave in evidence showing its role in migration and clinical outcome, such as what is described in “Helicobacter pylori infection facilitates cell migration and potentially impact clinical outcomes in gastric cancer”. Right now the background feel slightly procedural, and I think anchoring it more firmly in cancer biology would improve flow and relevance.

 In the methods, things are mostly clear, though some choices feel under-explained. I find myself wondering why certain thresholds and comparisons are treated as self-evident when they are not, especially for readers outside pathology. A bit more justification, not more detail but more reasoning, would help. The writing here is fine but feel slightly rushed, and tightening the logic rather than adding length would make it stronger.

 The results section present a lot of numbers and comparisons, and while the data appear consistent, the narrative interpretation drift a bit. At times it sound like performance differences are more decisive than they really are. I would recommend slowing down the interpretation and reminding the reader what question is actually being answered at each step. Some repetition is okay here, but clarity matter more. Right now it feel like the data talk faster than the conclusions can keep up.

 In the discussion, this is where most of the overreach happen. The study do something useful, but it do not yet redefine practice. I would strongly encourage the authors to separate what the data directly show from what they hope might be true in future settings. There is a tendency to generalize beyond the cohort and beyond the technical scope, and pulling that back would actually make the paper more credible. A bit more self-critique, especially around generalizability and clinical translation, would be welcome. It almost get there, but not quite.

Is the work clearly and accurately presented and does it cite the current literature?

Yes

If applicable, is the statistical analysis and its interpretation appropriate?

Yes

Are all the source data underlying the results available to ensure full reproducibility?

Yes

Is the study design appropriate and is the work technically sound?

Yes

Are the conclusions drawn adequately supported by the results?

Yes

Are sufficient details of methods and analysis provided to allow replication by others?

Yes

Reviewer Expertise:

cancer

I confirm that I have read this submission and believe that I have an appropriate level of expertise to confirm that it is of an acceptable scientific standard, however I have significant reservations, as outlined above.

# Response

**Published:** 2026-01-05

**Authors:** Nasar Alwahaibi

**Affiliations:** Biomedical Science, Sultan Qaboos University, Muscat, Oman

We would like to take this opportunity to express our thanks to the reviewer for the positive feedback and helpful comments.

 Below are our responses, point-by-point to the queries of the reviewer.

 Reviewer comments

 In the abstract, the overall structure work fine but the confidence level feel a bit too high given what actually get tested. Right now it read close to a clinical conclusion, and that make me uneasy. I would really dial back the implication that this approach already meaningfully inform diagnosis or patient outcome, and instead frame it more clearly as a comparative methodological assessment with downstream relevance rather than immediate application. Shortening some claims would help. Just a little restraint here go a long way.

**Response**

**As suggested, we revised the Conclusions section of the abstract to use more cautious language. The findings are now presented as informing possible approaches and future research, rather than making immediate diagnostic. We also clarified that the study represents a methodological assessment and that further validation is needed before clinical use.**

 The background section need more grounding in disease biology and clinical context. Helicobacter pylori is not just a technical detection problem, it is a causal driver of gastric carcinogenesis and disease progression, and that connection should be stated clearly and early so the reader understand why this question matter beyond the lab bench. A brief discussion of how H. pylori infection contribute to gastric cancer development and behavior would strengthen the motivation a lot, and it would be very natural to weave in evidence showing its role in migration and clinical outcome, such as what is described in “Helicobacter pylori infection facilitates cell migration and potentially impact clinical outcomes in gastric cancer”. Right now the background feel slightly procedural, and I think anchoring it more firmly in cancer biology would improve flow and relevance.

**Response**

**As suggested, we have revised the Introduction section to clearly state *H. pylori's* causal link to gastric cancer development and its implications for disease progression and clinical outcomes. We have incorporated a brief discussion on how *H. pylori* infection contributes to gastric cancer, including a reference to the type of evidence highlighted in your comment (e.g., its role in cell migration and clinical outcomes).**

 In the methods, things are mostly clear, though some choices feel under-explained. I find myself wondering why certain thresholds and comparisons are treated as self-evident when they are not, especially for readers outside pathology. A bit more justification, not more detail but more reasoning, would help. The writing here is fine but feel slightly rushed, and tightening the logic rather than adding length would make it stronger.

**Response**

**We appreciate your comment regarding the Methods section. We agree that providing more justification and reasoning for certain choices enhances clarity, especially for non-specialist readers.**

**As suggested, we have added brief explanations for why Giemsa was selected as the reference standard and provided context for the chosen AUC interpretation thresholds. We also reinforced the rationale behind blinding and expert review to enhance the logical flow and address any perception of the writing being rushed.**

 The results section present a lot of numbers and comparisons, and while the data appear consistent, the narrative interpretation drift a bit. At times it sound like performance differences are more decisive than they really are. I would recommend slowing down the interpretation and reminding the reader what question is actually being answered at each step. Some repetition is okay here, but clarity matter more. Right now it feel like the data talk faster than the conclusions can keep up.

**Response**

**As suggested, we have carefully re-examined the Results section. We have focused on slowing down the interpretation, explicitly linking each finding back to the study's core questions (e.g., assessing H&E and AB/PAS as alternatives to Giemsa), and using more tempered language when discussing performance differences.**

 In the discussion, this is where most of the overreach happen. The study do something useful, but it do not yet redefine practice. I would strongly encourage the authors to separate what the data directly show from what they hope might be true in future settings. There is a tendency to generalize beyond the cohort and beyond the technical scope, and pulling that back would actually make the paper more credible. A bit more self-critique, especially around generalizability and clinical translation, would be welcome. It almost get there, but not quite.

**Response**

**As suggested, we have thoroughly revised the Discussion to address these points. We have focused on strengthening the self-critique regarding generalizability and clinical translation, ensuring that the interpretations are grounded explicitly in the data presented.**

**Thank you.**

# Reviewer report: Reviewer response for version 1

**DOI:** 10.5256/f1000research.187732.r429654

**Published:** 2025-11-25

**Authors:** Nicoleta Gimiga

**Affiliations:** 1 Grigore T. Popa” University of Medicine and Pharmacy, Iasi, Romania

The manuscript presents a large retrospective analysis comparing three widely used histological stains—H&E, Giemsa, and AB/PAS—for the detection of *Helicobacter pylori* in gastric biopsies. The topic is relevant, as histological diagnosis remains a cornerstone in *H. pylori* detection, and the study addresses a practical gap concerning diagnostic performance and cost-effectiveness.

 Strenghts:

 - **Large sample size **The inclusion of **816 biopsy cases** is a major strength and provides statistical reliability and generalizability.

**Direct comparison of three staining methods**-Few studies offer such a direct and comprehensive comparison.

 -Addresses a genuine gap in routine diagnostic pathology.

**Blind assessment-**The use of three independent examiners helps minimize observer bias.

 LIMITATIONS:

 Retrospective design

 Use of Giemsa as the sole “gold standard”

 The manuscript is **promising and clinically relevant**, with strong methodological foundations and valuable practical implications.

Is the work clearly and accurately presented and does it cite the current literature?

Yes

If applicable, is the statistical analysis and its interpretation appropriate?

Yes

Are all the source data underlying the results available to ensure full reproducibility?

Yes

Is the study design appropriate and is the work technically sound?

Yes

Are the conclusions drawn adequately supported by the results?

Yes

Are sufficient details of methods and analysis provided to allow replication by others?

Yes

Reviewer Expertise:

Pediatrics, Pediatric gastroenterologyst

I confirm that I have read this submission and believe that I have an appropriate level of expertise to confirm that it is of an acceptable scientific standard.
